# Analysis of TabZIP15 transcription factor from *Trichoderma asperellum* ACCC30536 and its function under pathogenic toxin stress

**DOI:** 10.1038/s41598-020-72226-w

**Published:** 2020-09-15

**Authors:** Zeyang Yu, Zhiying Wang, Yuzhou Zhang, Yucheng Wang, Zhihua Liu

**Affiliations:** 1 School of Forestry, Northeast Forestry University, No. 26 Hexing Road, Harbin, 150040 China; 2 College of Forestry, Shenyang Agricultural University, Shenyang, 110866 China; 3 General Station of Forest and Grassland Pest Management, National Forestry and Grassland Administration, No. 58 Huanghe Street, Shenyang, 110034 China

**Keywords:** Microbiology, Fungi

## Abstract

The *TabZIP15* gene encoding a 396 amino acid (aa) polypeptide in the fungus *Trichoderma asperellum* ACCC30536 was cloned and characterised. The protein includes a basic region motif (NR-x2-QR-x2-R) and has a pillar-like structure. The 25 basic region/leucine zipper transcription factors (TFs) identified in the *T. asperellum* genome were divided into YAP (14 TFs), ATF2 (5), GCN4 (2), Zip1 (2), BRLZ (1) and u1 (1) subfamilies based on conserved domains. *T. asperellum* was cultured in minimal media (MM) control, C-Hungry and N-Hungry medium (to simulate nutrient competition and interaction with pathogens, respectively), and differential expression analysis showed that 14 *TabZIP* genes (including *TabZIP15*) were significantly altered under both conditions; *TabZIP23* responded strongly to N-Hungry media and *TabZIP24* responded strongly to C-Hungry media. However, only YAP genes *TabZIP15*, *TabZIP12* and *TabZIP2* were significantly upregulated under both conditions, and expression levels of *TabZIP15* were highest. *T. asperellum* was also cultured in the presence of five fungal pathogenic toxins, and RT-qPCR results showed that *TabZIP15* was significantly upregulated in four of the five toxin stress conditions (MM + *Rhizoctonia solani*, MM + *Fusarium oxysporum*, MM + *Alternaria alternata* and MM + *Cytospora chrysosperma*).

## Introduction

Members of the fungal genus *Trichoderma* are important biological control agents, and their biological control mechanisms have been investigated in detail^1–3^. Species in the *Trichoderma* genus can readily adapt to their environment, propagates and grow, hence their use worldwide. Genome sequencing programs have targeted *Trichoderma*^4^ and the genomes of 16 *Trichoderma* species have been published (https://mycocosm.jgi.doe.gov/mycocosm/home/), laying the foundation for molecular biological analysis.

Basic region/leucine zipper (bZIP) transcription factors (TFs) are one of the largest and most diverse TF families, and proteins with bZIP domains are present in all eukaryotes^5^. These proteins possess two distinctive structural features located on a contiguous alpha helix; (1) a basic region of ~ 16 amino acid (aa) residues comprising a nuclear localisation signal followed by an invariant N-x7-R/K motif that is responsible for interacting with DNA and nuclear import, and (2) a heptad repeat of leucines or other bulky hydrophobic amino acids positioned exactly nine amino acids from the C-terminus that creates an amphipathic helix which regulates dimerisation^5^. The DNA binding and dimerisation mechanism in fungi has been studied in *Saccharomyces cerevisiae*, and the results revealed that Met4 and Met28 form a heteromeric complex with a centromere binding factor to activate transcription^6,7^.

The mechanism of bZIP TFs has been analysed in various fungus species^8^. Recent studies showed that members of the bZIP TF family play diverse regulatory roles in filamentous fungi. Eight bZIP TFs in *Ustilaginoidea virens* are involved in stress tolerance and pathogenicity^9^. In *Aspergillus* spp., bZIP TFs including AtfA, NapA, AflR, RsmA and Apyap1 have been demonstrated to be the key factors responding to oxidative, osmotic, environmental and drug stresses^10–13^. In other fungi, such as *Botrytis cinerea*, *Neurospora crassa*, *Fusarium graminearum* and *Magnaporthe oryzae*, bZIP TFs are known to be involved in responding to oxidative stress and pathogenicity^14,15^. These findings suggest that bZIP TFs play an important role in stress responses in several fungi, and a similar regulatory function can be inferred in other fungal species. However, most of the research on fungal bZIP proteins has focused on pathogens, which bZIP proteins in biological control agents such as *Trichoderma* spp. have received little attention. Phytopathogen toxins are metabolites in pathogens that can harm plants and other fungi. To better understand the detoxification mechanism of *Trichoderma* that is triggered in response to phytopathogen toxins, the roles of bZIP TFs should be investigated under different stress conditions.

Herein, the *TabZIP15* TF gene of *Trichoderma asperellum* ACCC30536 was cloned, and characterised alongside another 24 similar bZIP TFs in the Joint Genome Institute (JGI) database. The basic biochemical characteristics of these bZIP TFs were investigated, and expression levels were analysed based on RT-PCR data obtained for *T. asperellum* under three conditions at 72 h. Furthermore, transcription levels of *TabZIP15* were measured by RT-qPCR following exposure to toxins produced by five pathogenic fungi (*Rhizoctonia solani*, *Fusarium oxysporum*, *Sclerotinia sclerotiorum*, *Alternaria alternata*, and *Cytospora chrysosperma*). The results provide theoretical support for the analysis and development of TFs from *T. asperellum*.

## Materials and methods

### Strains and materials

*T. asperellum* ACCC30536 was obtained from the Agricultural Culture Collection of China. The five phytopathogens were *R. solani*, *F. oxysporum*, *S. sclerotiorum*, *A. alternata* and *C. chrysosperma*, and they were stored at the Laboratory of Forestry Protection, Northeast Forestry University, Harbin, China. *T. asperellum* was cultured on potato dextrose agar (PDA) slant culture medium at 28 °C for 7 days and stored at 4 °C. *A. alternata*, *S. sclerotiorum*, *R. solani*, *C. chrysosperma* and *F. oxysporum* were inoculated in 200 mL of 1/4, 1, 1/2, 1 and 1/2 strength PD medium and cultured in shake flasks for 10 days at 28 °C with shaking at 200 rpm. The medium was filtered using 0.2 μm filters (Pall Corporation, MI, USA) to remove spores, and the filtered fermentation liquid from each pathogen was combined, mixed, and stored in tubes at − 20 °C.

### Cloning and analysis of *TabZIP15* transcription factor from *T. asperellum*

Primers for cloning *TabZIP15* (TabZIP15-1, 5′-GCGAATCCGGATGAGTTCAC-3′; TabZIP15-2, 5′-TCGCCGCACCCTATACTTTT-3′) were designed using Primer Premier 6.0 software (PREMIER Biosoft, Vancouver, Canada)^16^. Extraction of *T. asperellum* DNA was performed as described previously^17^, and thermal cycling for PCR was performed at 94 °C for 5 min, followed by 35 cycles at 94 °C for 25 s, 56 °C for 30 s, and 72 °C for 30 s, and followed by 72 °C for 7 min. The PCR product was purified and ligated into the pGEM-T vector (A3600; Promega, Madison, USA) and sequenced (Shanghai Sangon Co., Shanghai, China). Conserved domain prediction and identification of other homologous proteins was performed using the Blastp tool^18^ from the National Center for Biotechnology Information (NCBI). Multiple sequence alignment was conducted using the Clustal X program (https://www.ebi.ac.uk/Tools/clustalw2/)^19^, and three-dimensional structure prediction was carried out by SWISS-MODEL ( https://swissmodel.expasy.org/)^20^.

### Characteristic analysis and construction of phylogenetic tree of 25 bZIPs in the *T. asperellum* genome

Full-length *T. asperellum* bZIP protein (TabZIP protein) sequences were obtained from the *T. asperellum* CBS 433.97 v1.0 JGI database (https://genome.jgi.doe.gov/)^21^, and conserved domains were predicted using the NCBI tool Blastp. Proteins without conserved bZIP domains were omitted. The molecular weight (MW) and isoelectric point (pI) of each bZIP protein was calculated using ExPASy (https://www.expasy.org/)^22^, and gene structural information for exons and introns was obtained from the JGI database (https://genome.jgi.doe.gov/)^21^ and analysed by the Gene Structure Display Server 2.0 website (https://gsds.cbi.pku.edu.cn/index.php)^23^. A phylogenetic tree was constructed via the maximum likelihood method with 1,000 bootstrap replicates using the MEGA 7.0 program^24^. Motifs were searched using the MEME suite (http://meme-suite.org/tools/meme)^25^ with the site distribution set as ‘zoops’, and the number of motifs set as ‘3’. Conserved domains and motifs were analysed by the MEME suite and TBtools^26^.

### Differential expression of *bZIP *family genes and expression of *TabZIP15* genes in the presence of five pathogenic toxins

Minimal media (MM) contained 15 g/L NaH_2_PO_4_, 5 g/L (NH_4_)_2_SO_4_, 600 mg/L CaCl_2_·2H_2_O, 600 mg/L MgSO_4_·7H_2_O, 5 mg/L FeSO_4_, 2 mg/L CoCl_2_, 1.6 mg/L MnSO_4_, 1.4 mg/L ZnSO_4_ and 0.1% (w/v) glucose. MM + A comprised MM plus 5% (v/v) fermentation liquid from *A. alternata*. MM + S comprised MM plus 5% (v/v) fermentation liquid from *S. sclerotiorum*. MM + R comprised MM plus 5% (v/v) fermentation liquid from *R. solani*. MM + C comprised MM plus 5% (v/v) fermentation liquid from *C. chrysosperma*. MM + F comprised MM plus 5% (v/v) fermentation liquid from *F. oxysporum*, C-Hungry was MM without a carbon source, and N-hungry was MM without a nitrogen source. Differential expression of 25 *bZIP* family genes was analysed using *T. asperellum* ACCC30536 transcriptome data obtained previously^27^, and the heatmap was drawn by TBtools^26^. Expression of *TabZIP15* genes was analysed by RT-qPCR under five pathogenic toxin stress conditions. Spores from *T. asperellum* were inoculated into 200 mL 1/4 strength PD broth medium at a final concentration 1 × 10^4^ spores/mL and cultured at 28 °C with continuous shaking at 200 rpm for 48 h. The resulting mycelia were filtered, washed, transferred to MM for 2 h, then transferred into medium containing different pathogenic toxins for 72 h, three replicates were set. Mycelia were collected at 0, 4, 8, 12, 24, 48, and 72 h. At each time point, the biomass was calculated (averaged from three replicates), and 25 mL mycelium cultures were collected and stored at − 80 °C. Total RNA was extracted from mycelia^28^ using TRIzol reagent (Invitrogen, Carlsbad, USA), digested with DNaseI (Promega, Madison, USA), and reverse-transcribed into cDNA using a PrimeScript RT Kit (TaKaRa, Dalian, China) according to the manufacturer’s instructions.

Expression levels of *TabZIP15* in *T. asperellum* were measured by RT-qPCR, and calculated according to the 2^−ΔΔCt^ method^29^ using cDNA as template, with *actin*, *α-tubulin* and *β-tubulin* as reference genes. Three RT-qPCR replicates were performed per cDNA sample. Primers for RT-qPCR (Table 1) were designed using Primer Premier 6.0 software (PREMIER Biosoft, Vancouver, Canada)^16^, and figures were drawn using Origin 8.0 (OriginLab Corp, Northampton, Massachusetts, USA)^30^.

**Table 1:** Primers for RT-qPCR.

Gene name	Primers	Sequences (5′–3′)	Tm (°C)	Product size (bp)
*TabZIP15*	Ta-bZIP 15-L	TGACCCTGACGCTCCTTTACCT	59.1	230
	Ta-bZIP 15-R	GAGGCATGAGACGCTCCATACG	59.3	
*α-tubulin*	αtu-L	CACATGGTTGACTGGTGCCCTA	58.6	240
	αtu-R	CTCGCCCTCTTCCATACCCTCT	59.0	
*β-tubulin*	βtu-L	CAAACCGCCCTGTGCTCCAT	59.0	245
	βtu-R	TCGGCTGAGGCATCCTGGTAT	58.9	
*actin*	Act-L	AGGCAACCTTCTCGCCAACG	59.0	256
	Act-R	TCGCTTCTCGACAATGCCAACT	58.9	

### Statistical analysis

All experimental data were subjected to analysis of variance (ANOVA) and independent sample *t* tests using SPSS19.0^31^. Statistical significance of differences between groups was determined by Turkey’s multiple range tests (*p* < 0.05).

## Results

### Characteristics of 25 TabZIP TFs in the *T. asperellum* genome

**Figure 1:**
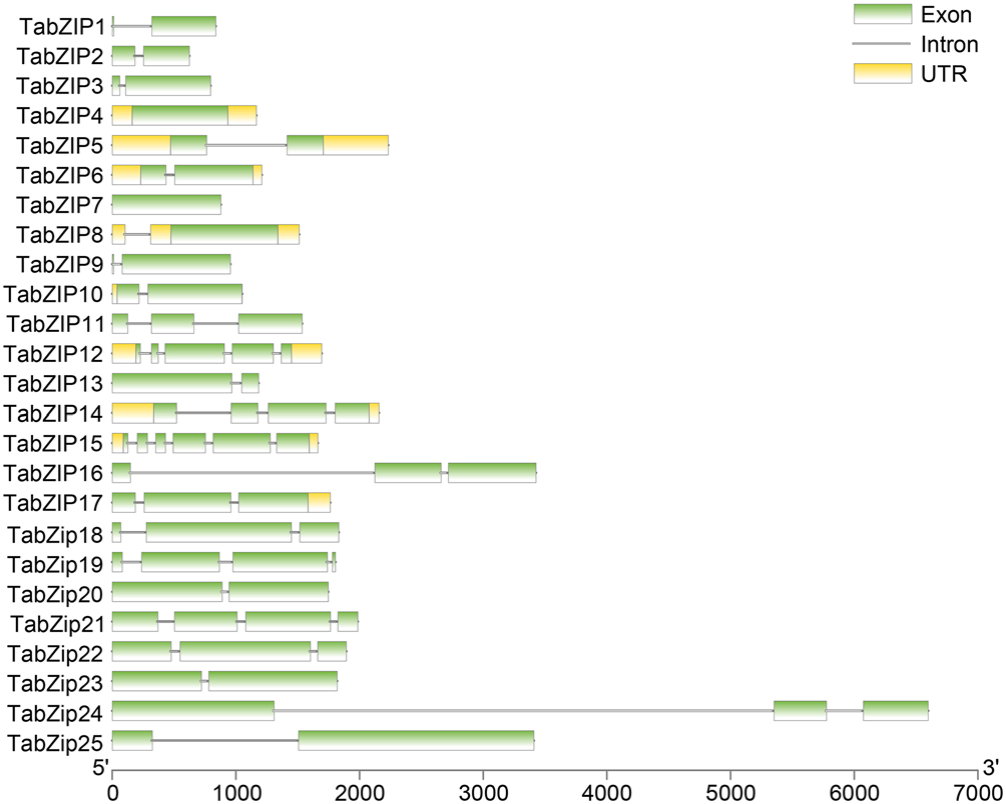
Gene structure of the 25 basic region/leucine zipper genes identified in *Trichoderma asperellum* (*TabZIP* genes). Green rectangles represent coding regions, yellow rectangles represent untranslated regions (UTRs), and lines represent introns; The x-axis shows the number of base pairs.

The 25 *TabZIP* genes encode proteins ranging from 177 to 753 aa in length, with an average length of 408 aa. The molecular weight of TabZIP proteins ranges from 19.63 to 80.93 kDa, with an average of 44.7 kDa. The pI ranges from 4.66 to 10.05, and the average pI is 6.65. *TabZIP* TF genes in *T. asperellum* are distributed on scaffold 1 to scaffold 41, and some chromosomes have more than one *TabZIP* TF gene (four, three and three TFs are in scaffold 5, scaffold 2 and scaffold 9, respectively; Table 2). The number of exons in the 25 *TabZIP* genes ranges from one to six, with most having two or three exons. Eight genes possess untranslated exon regions (Fig. 1).

**Table 2:** Characteristics of bZIP transcription factors in *Trichoderma asperellum*.

Gene name	Transcript ID	Gene location	Strand	AA	pI	MW
*TabZIP1*	72028	scaffold_12:344,401–345,238	+	177	6.87	19.63
*TabZIP2*	153231	scaffold_20:130,630–131,253	+	185	6.33	20.81
*TabZIP3*	457151	scaffold_6:308,334–309,129	−	250	6.17	28.39
*TabZIP4*	63179	scaffold_41:25,650–26,815	+	258	6.11	28.99
*TabZIP5*	56358	scaffold_4:1,710,394–1,712,625	+	268	5.54	29.91
*TabZIP6*	55919	scaffold_4:345,016–346,225	+	279	8.72	31.14
*TabZIP7*	70440	scaffold_9:580,536–581,414	+	293	6.12	31.72
*TabZIP8*	399278	scaffold_6:822,571–824,082	−	288	5.39	31.83
*TabZIP9*	48449	scaffold_9:461,415–462,370	−	296	8.43	32.56
*TabZIP10*	173677	scaffold_11:495,417–496,467	−	313	7.1	33.71
*TabZIP11*	43359	scaffold_2:1,460,822–1,462,357	+	328	5.95	36.03
*TabZIP12*	163644	scaffold_4:1,703,975–1,705,668	−	329	7.89	36.42
*TabZIP13*	26352	scaffold_5:2,050,878–2,052,061	−	368	8.48	40.11
*TabZIP14*	211303	scaffold_1:919,442–921,598	+	382	6.71	41.55
*TabZIP15*	88859	scaffold_5:1,544,405–1,546,067	−	396	5.54	45.05
*TabZIP16*	24989	scaffold_4:290,096–293,522	−	465	7.21	50.6
*TabZIP17*	192727	scaffold_6:1,483,125–1,484,887	+	483	5.84	53.93
*TabZip18*	29180	scaffold_10:1,049,874–1,051,705	−	520	9.21	54.65
*TabZip19*	28480	scaffold_9:647,376–649,180	−	501	6.04	55.97
*TabZip20*	141914	scaffold_8:846,956–848,702	+	564	4.66	61.51
*TabZip21*	31164	scaffold_15:854,551–856,536	−	575	5.54	63.22
*TabZip22*	65816	scaffold_3:815,417–817,309	+	588	5.09	64.07
*TabZip23*	134013	scaffold_4:888,003–889,821	+	587	4.97	64.56
*TabZip24*	23837	scaffold_2:2,610,317–2,616,914	−	753	6.22	80.43
*TabZip25*	22310	scaffold_1:981,806–985,215	−	743	10.05	80.93

*AA* number of amino acids, *pI* isoelectric point, *MW* molecular weight.

### Phylogenetic analysis, conserved domains, and motif prediction

**Figure 2:**
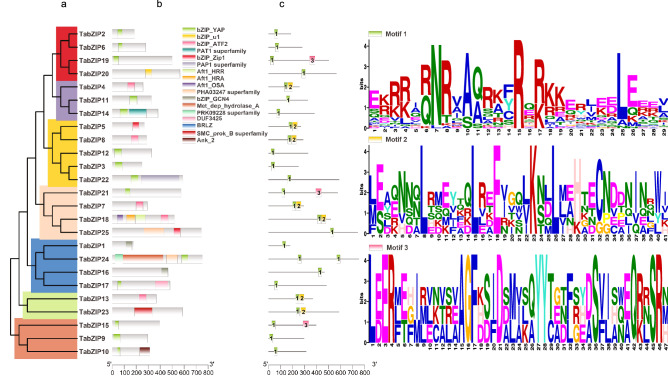
Phylogenetic relationships, conserved domain prediction, and motif composition of the 25 bZIP transcription factors (TFs) indentified in *T. asperellum* (TabZIP TFs). (**a**) Phylogenetic relationships among the 25 TabZIP TF amino acids; (**b**) Conserved domain prediction using the NCBI database. The x-axis shows amino acid numbers; (**c**) Motif prediction. The x-axis shows amino acid numbers.

Phylogenetic relationships between the 25 TabZIP proteins were explored, and sequences were divided into seven clades (Fig. 2a). The number of conserved domains in TabZIP TFs ranges from one to four (Fig. 2b), with seven bZIP TFs (TabZIP10, TabZIP17, TabZIP18, TabZIP22, TabZIP23, TabZIP24 and TabZIP25) having more than one conserved domain. Fourteen bZIP TFs have a bZIP-YAP conserved domain, five have a bZIP-ATF2 conserved domain, two have a bZIP-GCN4 conserved domain, two have a bZIP-Zip1 conserved domain, one has a BRLZ conserved domain, and one has a bZIP-u1 conserved domain. The 25 bZIP TFs were searched for three motifs (Fig. 2c), Motif 1 (E[KR][RK][RK][AI][QR]NR[VA]A[QA]R[KA][FYC]R[QE][RK][KR]KER[LI][EK][ED]LEEE[LV]); Motif 2 ([LM][ES]A[EQ]NN[NQ]LRMEYXQLREE[IV]GQ[LV]K[NS][DL]L[LM][AE]HTECNDDNINXWI); and Motif 3 (L[ED]ER[FLM][ET][FGH][IM][LMR][ELV][CKN][ATV][LRS][AEV]AGF[DHK][SD][IF]D[ADS][ML][AIV][KLS][QA]YY[TC][AEG][DNT][FL][GRS][EHY][DA]S[VF][IL][AHS][NQW][EA]Q[RK][LNR]SR[HMN]). We found that 10 bZIP TFs have two motifs, but interestingly, none of the bZIP TFs have three motifs and bZIP TFs in the same clade have similar numbers and types of motifs (Fig. 2).

### Differential expression of 25 *TabZIP*s in the three transcriptomes

**Figure 3:**
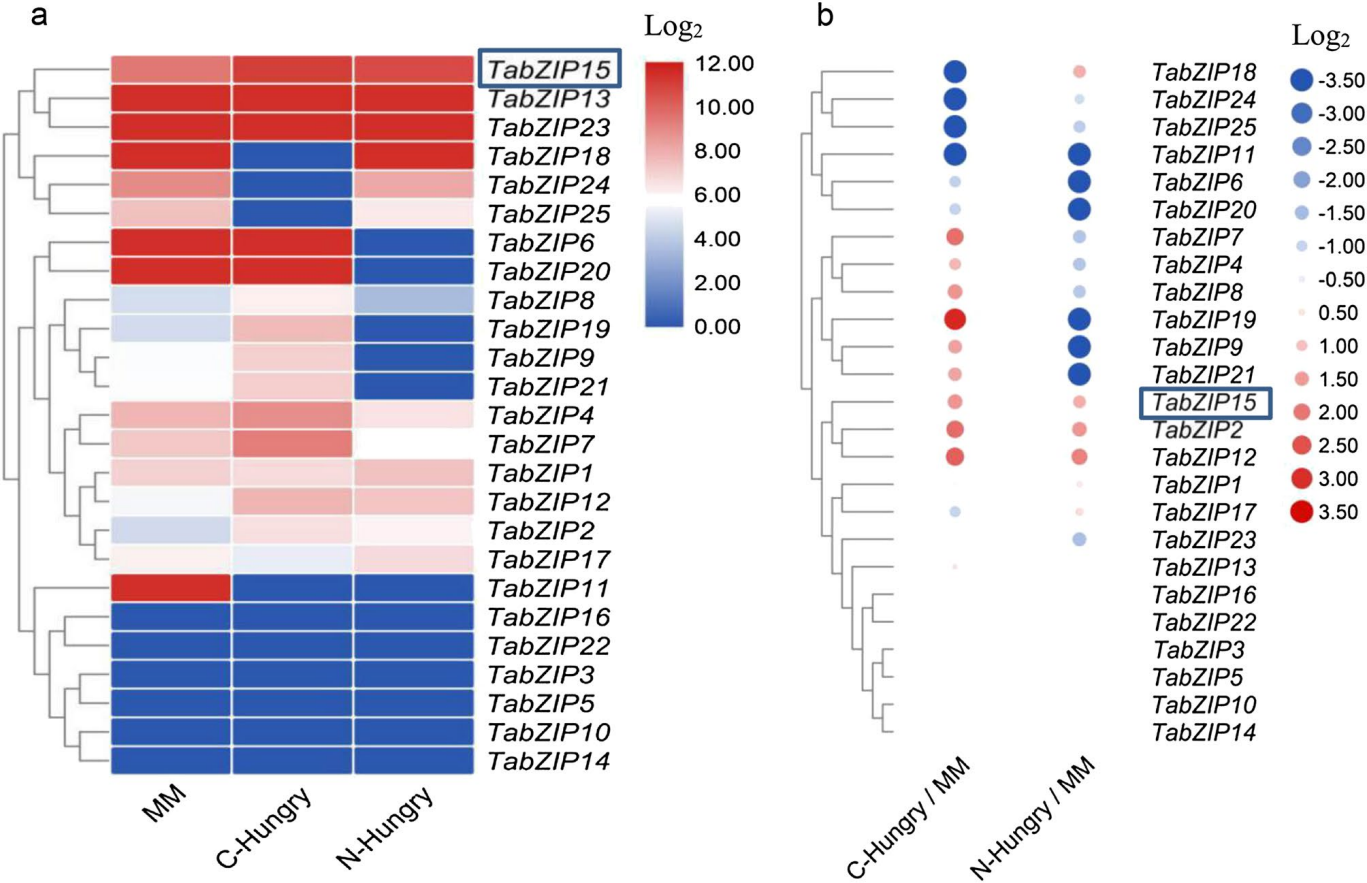
Heatmaps of the expression of the 25 *TabZIP* TF genes. (**a**) Expression levels of 25 *TabZIP* genes in three transcriptomes. (**b**) Differential expression of the 25 *TabZIP* genes (values = Log_2_(Treatment/MM)).

Expression of the 25 *TabZIP* genes in 3 transcriptomes was measured (Fig. 3a; Supplementary Table S1). Interestingly, 13, 14 and 11 *TabZIP* genes were highly expressed (Log_2_RPKM > 6, Reads Per Kilobase per Million mapped reads) in MM, C-Hungry and N-Hungry media, respectively. Genes were clustered based on their expression, and *TabZIP15*, *TabZIP13* and *TabZIP23* were highly expressed in all three conditions, while *TabZIP18*, *TabZIP24* and *TabZIP 25* were highly expressed in MM and N-Hungry media, and *TabZIP6* and *TabZIP20* were highly expressed in MM and C-Hungry media.

In the differential expression analysis, a two-fold (Log_2_ value > 1) change in expression level relative to MM was considered significant. The results showed that six *TabZIP* genes were significantly upregulated and six were significantly downregulated in C-Hungry media (Fig. 3b). Meanwhile, four *TabZIP* genes were significantly upregulated and 11 were significantly downregulated in N-Hungry media (Fig. 3b). Additionally, 14 genes were significantly altered in N-Hungry and C-Hungry media compared with MM media, but only three genes (*TabZIP15*, *TabZIP12* and *TabZIP2*) were significantly upregulated in both N-Hungry and C-Hungry media, while *TabZIP6*, *TabZIP11*, *TabZIP20* and *TabZIP25* were the only genes significantly downregulated in these two conditions. During the stress response process, upregulated genes may act as positive response factors. Thus, we focused on *TabZIP15*, *TabZIP12* and *TabZIP2*, and *TabZIP15* displayed the highest expression, hence this gene was subjected to further analysis.

### Characteristics of TabZip15 TF in *T. asperellum*

**Figure 4:**
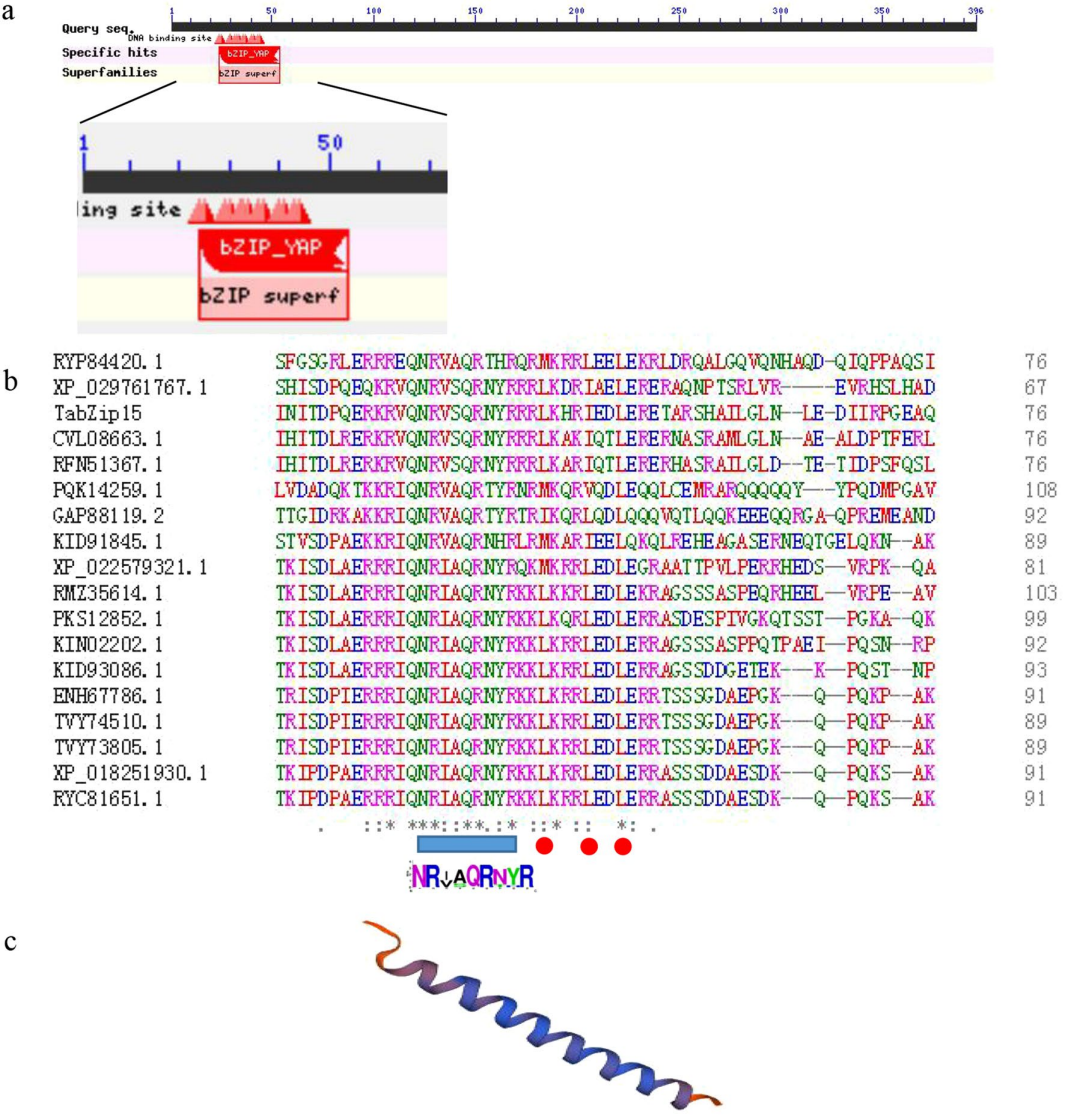
Characteristics of bZIP transcription factor TabZIP15. (**a**) Conserved domains in TabZIP15 predicted by NCBI Blastp. (**b**) Multiple sequence alignment of TabZIP15 and another 17 TFs. Except for TabZIP15, all names are NCBI accession numbers. Asterisk (*) represent identity, colon (:) represent high similarity, and periods (.) represent low similarity. Basic regions are marked by blue squares and hydrophobic amino acids are marked by red circles. The sequence logo was created based on the sequences of the basic region, and the height of letters in the logo represent the sequence conservation at that position. (**c**) Predicted three-dimensional structure of the TabZIP15 protein.

The *TabZIP15* DNA sequence (2062 bp) includes six exons and five introns. The *TabZIP15* cDNA (1,188 bp) encodes a 396 aa polypeptide with a calculated molecular weight of 45.05 kDa, and a pI of 5.54. TabZIP15 is a member of the bZIP-YAP family of TFs (Fig. 4a). Multiple sequence alignment revealed a highly conserved basic region motif (NR-x2-QR-x2-R), and three conserved hydrophobic amino acids in the sequence following the basic region (Fig. 4b). Three-dimensional structure prediction showed that the TabZIP15 protein is a cylindrical bundle of α-helices (Fig. 4c).

### Differential expression of *TabZIP15* under five fungal toxin stress conditions

**Figure 5:**
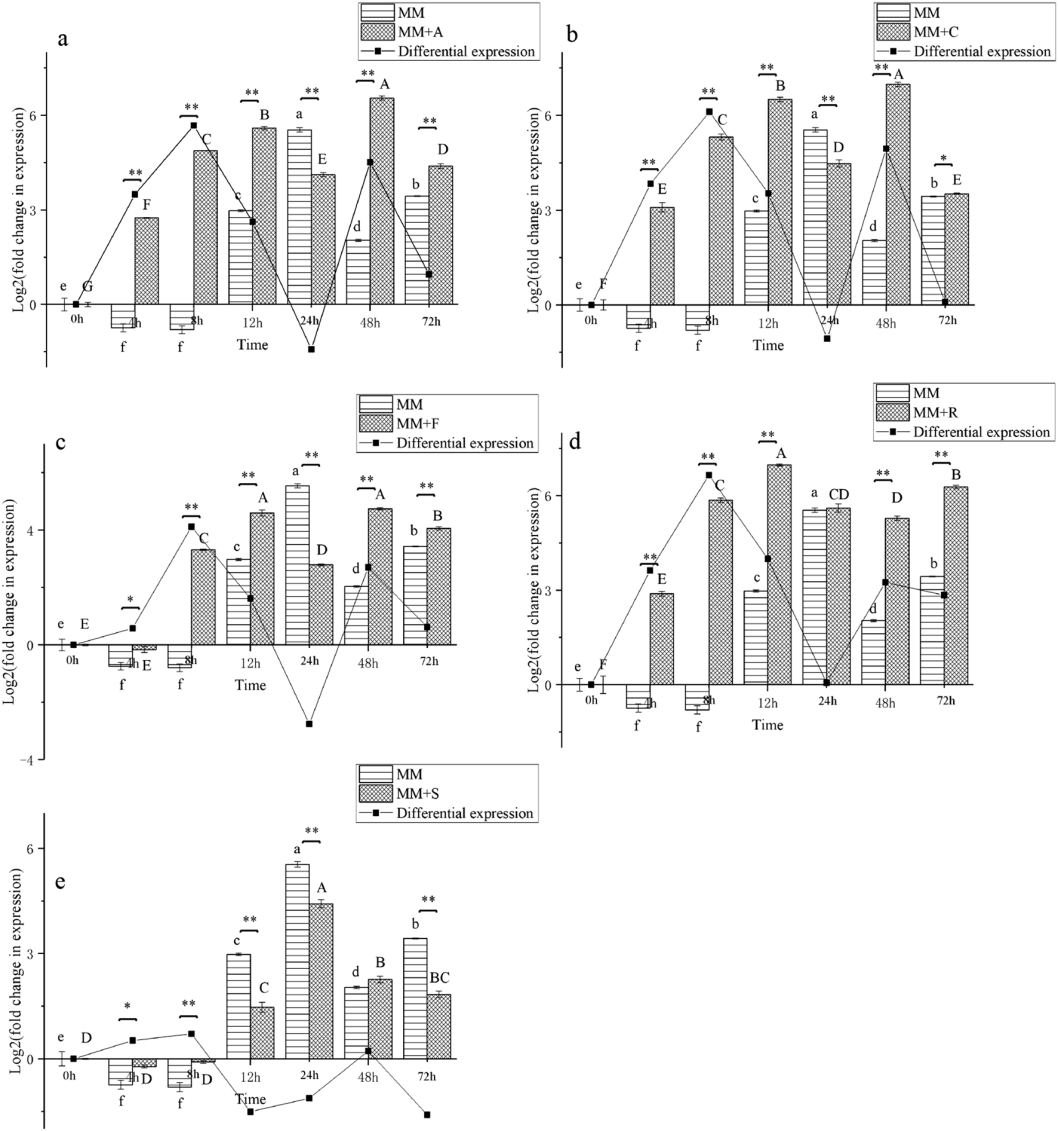
Expression of the *TabZIP15* gene in the presence of five fungal pathogenic toxins. Differential expression was measured between treatments, and minimal media (MM) alone served as a control. Error bars represent standard deviation. In each condition, different letters denote a statistically significant difference according to one-way analysis of variance (ANOVA; *p* < 0.05). Independent-sample *t* tests were performed on pairs of samples at the same timepoint (*p < 0.05; **p < 0.01, n = 3). (**a**) MM + A (*Alternaria alternata*). (**b**) MM + C (*Cytospora chrysosperma*). (**c**) MM + F (*Fusarium oxysporum*). (**d**) MM + R (*Rhizoctonia solani*). (**e**) MM + S (*Sclerotinia sclerotiorum*).

Expression of *TabZIP15* was investigated in the presence of five fungal pathogenic toxins (Fig. 5). In MM media, expression of *TabZIP15* was downregulated at 4 h and 8 h, then upregulated at 12 h, and the peak expression level (2^5.54^) occurred at 24 h. Expression of *TabZIP15* was upregulated at 4 h in MM + A, at 4 h in MM + C, at 8 h in MM + F, at 4 h in MM + R, and at 12 h in MM + S, with peak expression levels at 48 h (2^6.55^-fold), 48 h (2^6.98^-fold), 48 h (2^4.74^-fold), 12 h (2^6.98^-fold) and 24 h (2^4.42^-fold), respectively. Analysis of differential expression (relative to controls) showed that *TabZIP15* was highly expressed when *T. asperellum* was exposed to toxins from *A. alternata*, *C. chrysosperma*, *F. oxysporum* and *R. solani*, but expression in MM + S media did not differ significantly from that in MM media.

## Discussion

Members of the *Trichoderma* genus have been widely used as biological control agents, and the biocontrol mechanism has been investigated^3^. bZIP proteins regulate various biological processes^10–13^ and are present in all eukaryotes^5^. Thus, bZIP TFs may regulate biological control processes in *T. asperellum*. Herein, 25 TabZIP TFs were identified in *T. asperellum*, and multiple sequence alignment revealed relatively low sequence similarity (data not shown). Seventeen sequences sharing homology with TabZIP15 were identified using Blastp (Fig. 4), and multiple sequence alignment showed that these sequences share short conserved regions (~ 30 aa), as expected for bZIP proteins that typically include 18 conserved residues followed by a leucine zipper^32,33^. Thus, the sequences of bZIP TFs are diverse, and this may explain why they perform diverse functions^33^. The bZIP TFs in *T. asperellum* were divided into seven clades, with TabZIP15, TabZIP9 and TabZIP10 in the same clade (Fig. 2a). The close phylogenetic relationship indicates that these three proteins may perform similar functions and share similar expression profiles. Consistently, motif prediction showed that TabZIP15, TabZIP19 and TabZIP21 are the only proteins possessing motif 3, and they share a similar motif arrangement (Fig. 2c). This further suggests that TabZIP15, TabZIP19 and TabZIP21 may perform similar functions or share similar expression profiles.

Next, we explored the expression of these 25 *TabZIP* genes in C-Hungry and N-Hungry media using an RNA sequencing (RNA-Seq), which simulated *Trichoderma* nutrient competition and interaction with the pathogens^27^, TabZIP19 and TabZIP21 have similar motif arrangements to TabZIP15, hence they may be expected to share similar expression modes, but the RNA-Seq data showed that they did not have similar expression modes. Similarly, TabZIP9 and TabZIP10 are in the same clade as TabZIP15, and they share similar phylogenetic relationships, hence they may be expected to share similar expression modes, but again, RNA-Seq data showed that they did not have similar expression modes (Fig. 3).

The YAP protein and its homologs have been analysed in several fungal pathogens, and they all perform similar functions in stress response^9,15,34^. In *T. asperellum*, 14 of the 25 bZIP TFs have a conserved bZIP-YAP domain (Fig. 2b). The presence of a large number of YAP bZIPs may explain the robust ability of *T. asperellum* to adapt to environmental changes, but their exact functions need to be further investigated. In *Claviceps purpurea*, the transcription factor CPTF1, a homolog of ATF in mammals, is related to oxygen stress responses^35^. In *T. asperellum*, five bZIP proteins (TabZIP4, TabZIP7, TabZIP8, TabZIP13 and TabZIP18) have a conserved bZIP-ATF2 domain, and they may perform similar functions, but this should be further investigated (Fig. 2b). The TabZIP18 protein includes Aft1-HRR, Aft1-HRA and Aft1-OSA domains, and is a homolog of UvbZIP8^9^, AtfB^36^ and Aft1^37^. Aft1 proteins are reportedly involved in the regulation of sexual development and various stress responses, and they activate meiotic recombination, which suggests that TabZIP18 may also play a key role in these processes.

Our transcriptome data showed that 16 of the 25 *bZIP* TF genes were significantly differentially expressed in C-Hungry medium (simulating competition with pathogens) or N-Hungry medium (simulating interaction with pathogens; Fig. 2b). Of these, 15 genes appear to be involved in nutrient competition and 15 appear to be related to interactions with pathogens. The 15 nutrient competition-related genes include nine *YAP*, four *ATF2*, one *Zip1* and one *u1* gene*s*, while the 15 pathogen interaction-related genes include eight *YAP*, four *ATF2*, one *Zip1*, one *u1* and one *BRLZ* genes. These results suggest that YAP and ATF2 proteins in *T. asperellum* may be involved in various stress responses, similar to other homologs^9,15,34–37^. One *BRLZ* gene was significantly downregulated in N-Hungry media (interaction with pathogens), and a homolog of this protein is reportedly involved in fungal development and differentiation^38^. Meanwhile one *u1* and one *Zip1* were significantly downregulated in both N-Hungry and C-Hungry media. A homolog of *Zip1* was reportedly induced under stress conditions^39^, but *u1* proteins have not been investigated, and further studies are needed. Thirteen genes were highly expressed in MM media (Log_2_RPKM > 6), and they may be involved in normal physiological regulation (Fig. 2a). Three *YAP* genes (*TabZIP15*, *TabZIP12* and *TabZIP2*) were significantly upregulated in both C-Hungry and N-Hungry media (simulating nutrient competition and interactions with pathogens, respectively), which suggests that they may be important for resistance against pathogens. *TabZIP12* and *TabZIP2* were both significantly upregulated. *TabZIP12* expression levels were upregulated 2^2.31^- and 2^1.85^-fold in C-Hungry and N-Hungry media respectively, while *TabZIP2* expression levels were upregulated 2^2.14^- and 2^1.58^-fold in C-Hungry and N-Hungry media respectively. However, the RPKM value for *TabZIP12* in MM and C-Hungry media was 45 and 224, respectively; compared with 22 and 97 for *TabZIP2*. The RPKM value for *TabZIP12* in MM and N-Hungry media was 45 and 163, respectively, compared with 22 and 66 for *TabZIP2*. Thus, the RPKM values for both genes were not high, and can be easily influenced by experimental error^40^, but fold-changes in expression were relatively large, hence further investigation is clearly needed.

The most highly expressed gene (*TabZIP15*) was chosen for further analysis, and its expression was measured in the presence of five pathogenic toxins by RT-qPCR. The results confirmed our hypothesis (Fig. 5); *TabZIP15* was strongly upregulated in four of the five pathogenic toxin conditions, indicating an important function in resistance to pathogens. Interestingly, *TabZIP15* was also highly expressed in MM media, which suggests that it may be involved in other functions beyond resistance to pathogens.

Herein, we identified 25 *TabZIP* genes in *T. asperellum*, which were divided into seven clades based on phylogenetic analysis, and six conserved bZIP domains were detected in the aa sequences. Analysis of the transcriptome data showed that expression of 16 *TabZIP* genes was significantly altered under C-Hungry and N-Hungry media conditions. The *TabZIP15* gene was identified as a potential biocontrol factor, and its characteristics were further analysed. The RT-qPCR results showed that the *YAP* gene *TabZIP15* was significantly upregulated in the presence of four of the five pathogenic toxins (those from *A. alternata*, *C. chrysosperma*, *F. oxysporum* and *R. solani*). Our results reveal some of the properties of bZIP TFs in *T. asperellum*, identify potential biological control-related bZIP TFs, and will guide further research in this area.

## Supplementary information

Supplementary Table S1.

## Data Availability

All data generated or analysed during this study are included in this published article (and its Supplementary Information files), if there is any other request, the datasets are available from the corresponding author on reasonable request.
