# Supplementary material for: Analysis of TabZIP15 transcription factor from Trichoderma asperellum ACCC30536 and its function under pathogenic toxin stress
Source: Sci Rep. 2020 Sep 15;10:15084. doi: 10.1038/s41598-020-72226-w (PMC7493895; doi:10.1038/s41598-020-72226-w)
Supplement: Supplementary file 1 — Supplementary Table S1. [file 41598_2020_72226_MOESM1_ESM.pdf]

Analysis of TabZIP15 transcription factor from *Trichoderma asperellum* ACCC30536 and its function under pathogenic toxins stress

Zeyang Yu[1], Zhiying Wang[1], Yuzhou Zhang[3], Yucheng Wang[1, 2], Zhihua Liu[1 *, 2]
[1]School of Forestry, Northeast Forestry University, 26 Hexing Road, Harbin 150040, China

[2]College of Forestry, Shenyang Agricultural University, 110866, Shenyang, China

[3]Forest Disease and Pest Control Station of State Forestry Administration, Shenyang, China

EMAIL: Zeyang Yu: 645968312@qq.com, Zhiying Wang: 384162668@qq.com, Yuzhou Zhang: 790855708@qq.com, Yucheng Wang: 1019076345@qq.com, Zhihua Liu*: LZHNEFU@126.com
Tel: +86-451-82191512
Fax: +86-451-82190384

Supplementary Table 1. Expression levels of 25 *TabZIP* genes in 3 transcriptomes.

Name	Gene	MM (RPKM)	C-Hungry (RPKM)	N-Hungry (RPKM)
TabZIP1	fgenesh1_pm.12_#_105	128	106	176
TabZIP2	e_gw1.20.68.1	22	97	66
TabZIP3	MIX12808_1_13	0	0	0
TabZIP4	fgenesh1_kg.41_#_8_#_Locus11584v2rpkm0.00_PRE	221	476	91
TabZIP5	fgenesh1_kg.4_#_553_#_Locus823v1rpkm173.21	0	0	0
TabZIP6	fgenesh1_kg.4_#_114_#_Locus2525v1rpkm52.35	8054	3822	0
TabZIP7	fgenesh1_pm.9_#_200	152	650	61
TabZIP8	CE193182_966	24	73	10
TabZIP9	fgenesh1_pg.9_#_164	50	134	0
TabZIP10	estExt_Genewise1.C_11_t10386	0	0	0
TabZIP11	fgenesh1_pg.2_#_417	4164	0	0
TabZIP12	estExt_Genewise1.C_4_t30343	45	224	163
TabZIP13	gm1.4321_g	9006	11576	8249
TabZIP14	CE5207_15015	0	0	0
TabZIP15	estExt_fgenesh1_pg.C_5_t10463	732	2189	1742
TabZIP16	gm1.2958_g	0	0	0
TabZIP17	estExt_Genewise1Plus.C_6_t30146	70	35	107
TabZIP18	gm1.7149_g	3725	0	8729
TabZIP19	gm1.6449_g	23	197	0
TabZIP20	e_gw1.8.879.1	12083	5889	0
TabZIP21	gm1.9133_g	52	136	0
TabZIP22	fgenesh1_pm.3_#_271	0	0	0
TabZIP23	e_gw1.4.1538.1	9040	10069	3355
TabZIP24	gm1.1806_g	509	0	283
TabZIP25	gm1.279_g	177	0	80

Note: MM: minimal media; C-Hungry: MM without carbon source; N-hungry: MM without nitrogen source; RPKM: Reads Per Kilobase of exon model per Million mapped reads.
